# Type 2 diabetes alters quiescent pancreatic stellate cells to tumor-prone state

**DOI:** 10.1172/jci.insight.187424

**Published:** 2025-06-23

**Authors:** Yutaro Hara, Hiroki Mizukami, Takahiro Yamada, Shuji Shimoyama, Keisuke Yamazaki, Takanori Sasaki, Zhenchao Wang, Hanae Kushibiki, Masaki Ryuzaki, Saori Ogasawara, Hiroaki Tamba, Akiko Itaya, Norihisa Kimura, Keinosuke Ishido, Shinya Ueno, Kenichi Hakamada

**Affiliations:** 1 Department of Pathology and Molecular Medicine, Biomedical Research Center,; 2 Department of Gastroenterological Surgery, and; 3 Department of Neurophysiology, Biomedical Research Center, Hirosaki University, Hirosaki, Japan.

**Keywords:** Endocrinology, Gastroenterology, Cancer, Diabetes, Fibrosis

## Abstract

Pancreatic stellate cells (PSCs) are the origin of cancer-associated fibroblasts. Type 2 diabetes mellitus (T2D) may promote pancreatic ductal adenocarcinoma (PDAC), eliciting changes in the quiescent PSC (qPSC) population from the precancerous stage. However, the details are unknown. We evaluated the subpopulations of qPSCs and the impact of T2D. PSCs isolated from 8-week-old C57BL/6J mice and diabetic *db/db* mice were analyzed by single-cell RNA-seq. Sorted qPSCs and PDAC cells were transplanted into allogenic mice. The isolated qPSCs were broadly classified into mesothelial cell and pancreatic fibroblast (Paf) populations by single-cell RNA-seq. Pafs were subclassified into inflammatory Pafs, myofibroblastic Pafs (myPafs) and a small population named tumor immunity- and angiogenesis-promoting Pafs (tapPafs), expressing *Cxcl13*. In the subcutaneous transplantation model, the tumors transplanted with myPafs were significantly larger than the tumors transplanted with tapPafs. An increase in myPafs and a decrease in tapPafs were observed from the precancerous stage in human T2D, indicating the effects of tumor progression. This study revealed the subpopulation changes in qPSCs in T2D. A therapy that increases the number of tapPafs could be a therapeutic option for patients with PDAC and T2D and even those in a precancerous stage of T2D.

## Abstract 2

In type 2 diabetes mellitus, pancreatic stellate cells show a predominance of tumour-promoting trait from a pre-cancerous state and are involved in tumourigenesis of pancreatic ductal carcinoma.

## Graphical abstract

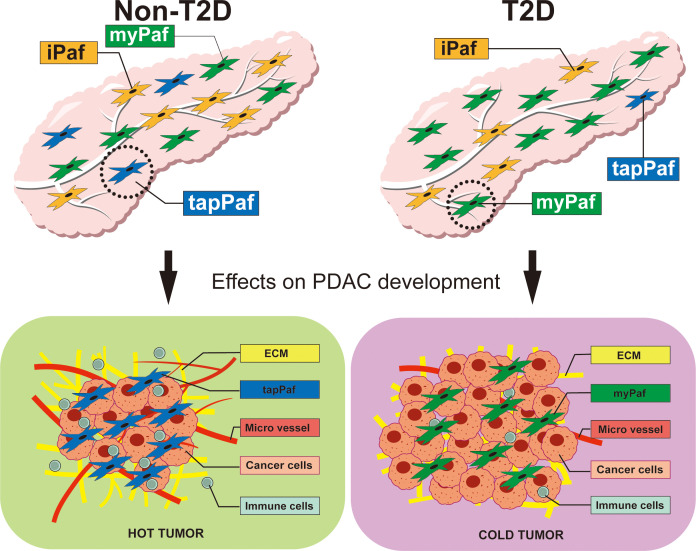

## Introduction

Invasive pancreatic ductal carcinoma (PDAC) is a solid tumor with one of the worst prognoses, with a 5-year survival rate of less than 10% (1). The incidence of PDAC has increased worldwide in recent years, and it is expected to become the second leading cause of cancer death by 2030 (2). Because no radical treatment has been established, exploring targets for new therapeutic mechanisms is urgently needed.

Pathologically, desmoplasia, which suppresses the immune response and angiogenesis and increases chemoresistance, is a hallmark of PDAC (3–5). Recently, it was revealed that the cancer stroma contains unique fibroblasts called cancer-associated fibroblasts (CAFs) (5–7). CAFs are composed of multiple cell populations and are classified into at least four cell populations: myofibroblastic CAFs (myCAFs), inflammatory CAFs (iCAFs), antigen-presenting CAFs (apCAFs), and mesenchymal stem cell-like CAFs (mscCAFs). Among CAFs, myCAFs expressing α-smooth muscle actin (α-SMA) and iCAFs expressing IL6 and other inflammatory cytokines are known to be poor prognostic factors (8, 9). Because apCAFs originating from mesothelial cells express MHC class II, apCAFs are considered to regulate antigen presentation in PDAC tissue (7, 10). mscCAFs, marked by Meflin expression, have been shown to improve the prognosis of patients with PDAC (11). Thus, CAF subpopulations play individual roles in tumor progression.

Pancreatic stellate cells (PSCs), which constitute 50% of the stroma, are involved in fibrosis of the pancreas (12, 13). Quiescent PSCs (qPSCs) are characterized by fat droplets in the cytoplasm and expression of FABP4 (14, 15), whereas activated PSCs, which are characterized by the expression of α-SMA, can produce large amounts of extracellular matrix (16). Although activated PSCs are assumed to be the origin of CAFs and account for the majority of myCAFs, the association of qPSCs with CAFs has not been well elucidated (5, 8, 17).

Type 2 diabetes mellitus (T2D) is known to be an oncogenic and poor prognostic factor for patients with PDAC (18–20). Our previous studies reported that a long duration of T2D (≥ 3 years) was correlated with a high frequency of *CDH1* promoter methylation and that a high level of HbA1c (≥ 6.5%) was correlated with high expression of miR-100-5p, resulting in low expression of E-cadherin, epithelial-mesenchymal transition, poor prognosis, and malignant behavior in patients with PDAC (21–23). Although we also reported that T2D could activate PSCs via TGF-β and IL6 signals (21), the impact of T2D on the diversity of qPSCs related to the CAF subpopulations in the precancerous stage remains unclear.

In this study, we explored the heterogeneity of qPSCs and the changes evoked by T2D using single-cell analysis. Our results revealed a mechanism of PDAC progression facilitated by T2D and may contribute to the development of novel therapeutic strategies for PDAC.

## Results

### Isolation of quiescent PSCs from WT and db/db mice.

**Figure 1:**
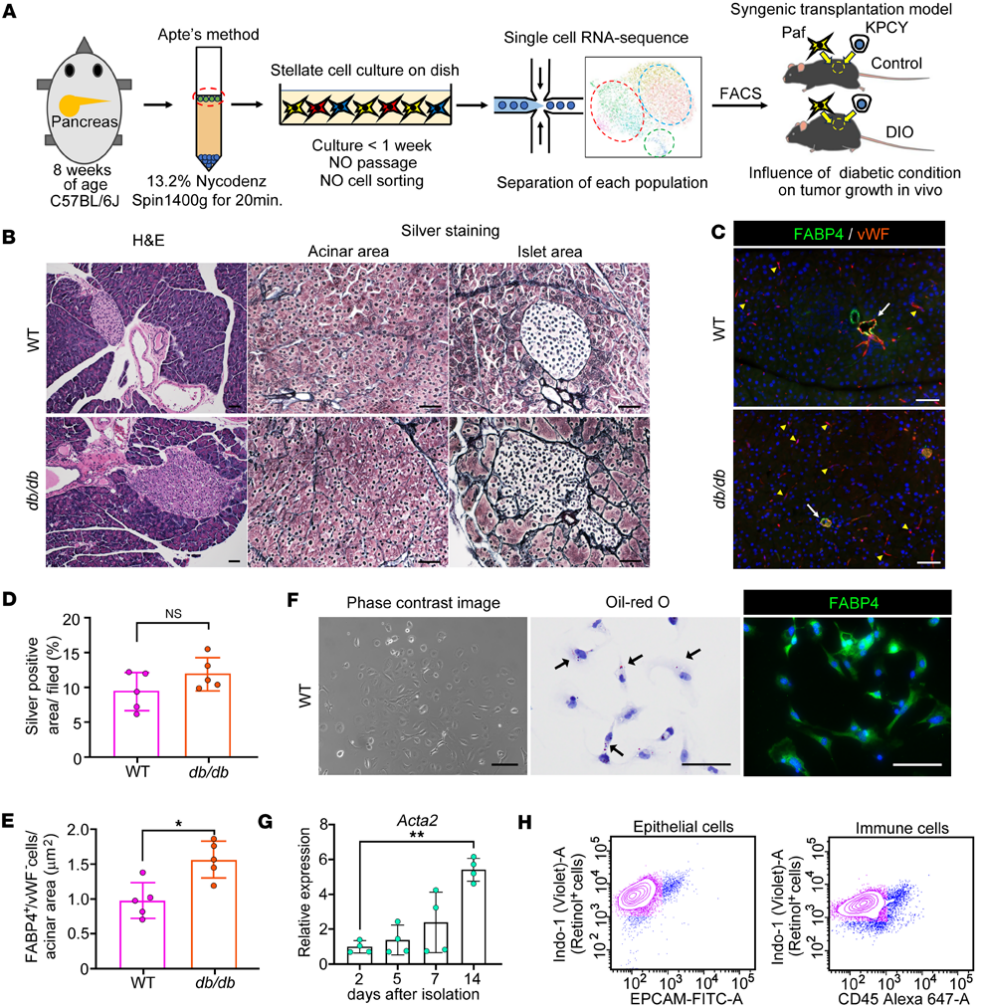
PSCs are quiescent in the pancreas of nondiabetic and diabetic mice. (**A**) Schematic overview depicting the approach used for the experiments. (**B**) H&E and silver staining showing pathological findings in the pancreas of WT and *db/db* mice (original magnification, ×10 and ×20). (**C**) Immunofluorescence image showing FABP4-positive and vWF-negative PSCs in the stroma of the pancreas from WT and *db/db* mice (original magnification, ×20). (**D**) Semiquantification of fibrosis in WT (*n* = 5) and *db/db* (*n* = 5) mice. (**E**) The density of FABP4-positive and vWF-negative cells in WT (*n* = 5) and *db/db* mice (*n* = 5). (**F**) Morphology of PSCs isolated from the pancreas. Lipid droplets detected by oil red O staining (arrows) in WT mice (*n* = 8). Immunofluorescence analysis of FABP4 expression in isolated PSCs (original magnification, ×20). (**G**) Time course of *Acta2* mRNA expression in PSCs after isolation evaluated by qPCR (*n* = 4 per each time point). (**H**) Representative flow cytometry plots showing retinol-positive cell clusters evaluated with indo-1 and EPCAM antibody, or indo-1 and CD45 antibody. The data are presented as the means ± SDs. Statistical analysis was performed by 2-way ANOVA with post hoc multiple-comparison tests. PSCs, pancreatic stellate cells; vWF, von Willebrand factor. ^*^P < 0.01 and ^†^*P* < 0.05. Scale bars: 100 μm (**B**, H&E staining) or 50 μm (others).

To understand the changes in the heterogeneity and function of qPSCs elicited by diabetic conditions, we performed droplet-based single-cell RNA-seq (scRNA-seq) on qPSCs isolated from female WT C57BL/6J mice (WT) and C57BLKS/J-m+/+*Lepr* *db* homozygous (*db*/*db*) mice (Figure 1A). Histological analysis of H&E-stained sections revealed that the islets of *db*/*db* mice were hyperplastic compared with those of WT mice (Figure 1B). Abnormal fibrosis, as evaluated by silver staining, was not prominent in the acinar area of either the WT or *db*/*db* mice, whereas periislet fibrosis was more evident in the *db*/*db* mice than in the WT mice (Figure 1B). Although the degree of fibrosis of the whole pancreas tended to be greater in *db*/*db* mice than WT mice, there was no significant difference between the two groups (Figure 1D). FABP4 is a known marker for qPSCs and endothelial cells (14, 15, 24). The density of FABP4-positive and von Willebrand factor–negative (vWF-negative) cells was significantly greater in *db*/*db* mice than in WT mice (Figure 1, C and E). Isolated and cultured qPSCs from WT mice presented a star-like morphology with lipid droplets that were positive for oil red O staining and FABP4 (Figure 1F). *Acta2* mRNA expression was comparable between 2 and 5 days after isolation, whereas the expression was significantly increased 6-fold 2 weeks after passaging (Figure 1G). Flow cytometry revealed that the percentage of retinol-positive cells was greater than 90%, while EPCAM-positive epithelial cells and CD45-positive immune cells were minimally contaminated (4.2% and 4.8%, respectively) (Figure 1H). These results confirmed that a large proportion of the isolated cells were qPSCs in WT mice, whereas a portion of the activated PSCs might be contained only in *db*/*db* mice.

### Heterogeneity of qPSCs revealed by scRNA-seq.

**Figure 2:**
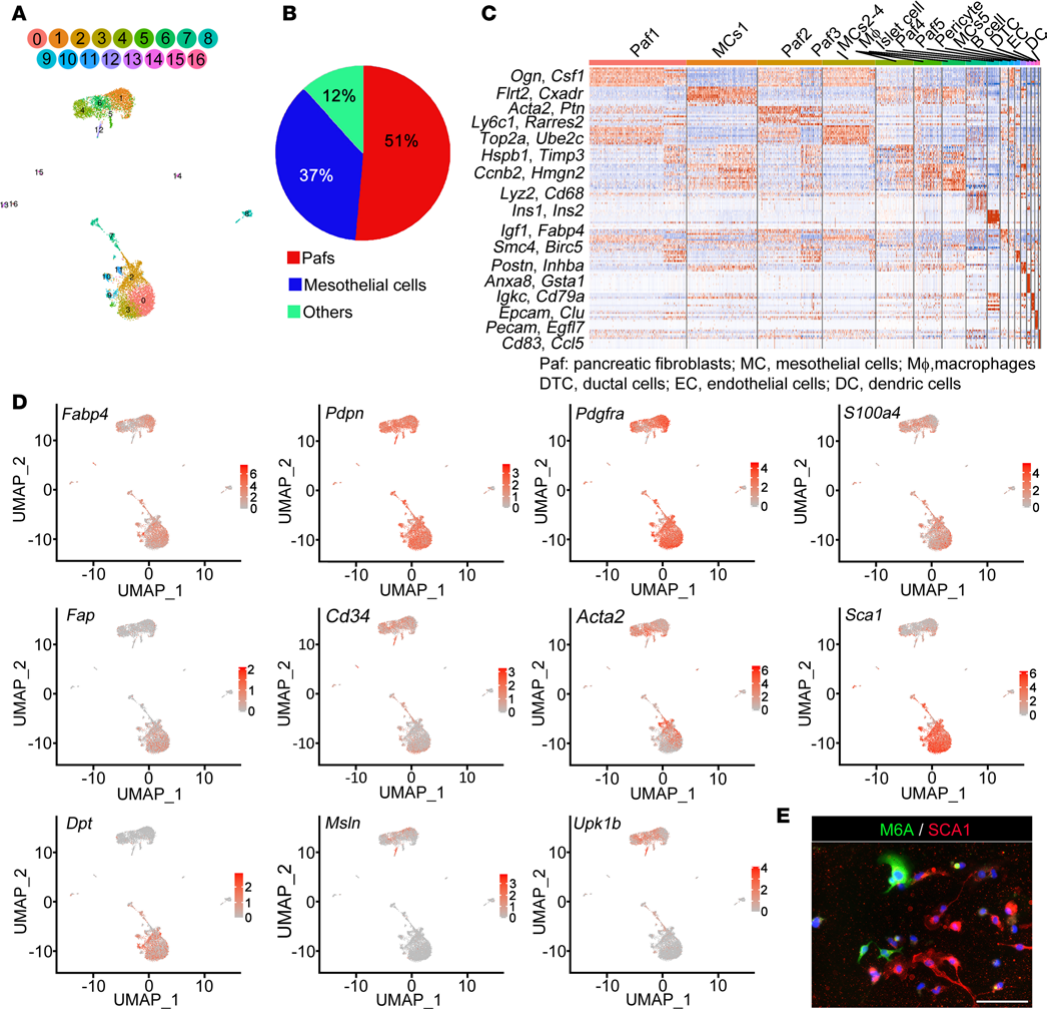
Heterogeneity of quiescent PSCs was revealed by scRNA-seq. (**A**) scRNA-seq revealed 16 cell subclusters of qPSCs isolated from the pancreas of nondiabetic mice via 2-dimensional UMAP (*n* = 2). (**B**) The populations of the isolated qPSCs are shown in the pie chart. (**C**) Heatmap of specific gene expression levels allowing subclassification of qPSCs into 16 cell groups. (**D**) UMAP plots showing the expression of signature genes of each cell type. (**E**) Representative double-immunofluorescence image illustrating the expression of M6A and SCA1 in isolated PSCs (original magnification, ×20). scRNA-seq, single-cell RNA sequence; qPSCs, quiescent pancreatic stellate cells; UMAP, uniform manifold approximation and projection; Pafs, pancreatic fibroblasts. Scale bar: 50 μm.

After quality control, 7,746 cells (4,932 cells from WT mice and 2,814 cells from *db*/*db* mice) with an average sequencing depth of 5,689 genes per cell (5,640 genes per cell from WT mice and 5,738 genes per cell from *db*/*db* mice) were subjected to further analysis via scRNA-seq. Unsupervised clustering classified these cells into 16 distinct clusters in WT mice (Figure 2A). The signature genes within each cluster were cross referenced with known markers of cell populations from the literature to identify the different cell types that are represented by the clusters (Figure 2B). Within the viable cell fraction, the majority of cells in our analysis were classified as pancreatic fibroblasts (Pafs) (51%), mesothelial cells (37%) or other cells (12%), which were further classified as macrophages, islet cells, pericytes, B cells, ductal cells, endothelial cells, or dendritic cells based on the expression of cell type–specific marker genes. Clusters 0, 2, 3, 9, and 10 were identified as Pafs on the basis of their expression of signature fibroblast genes (Figure 2C). Clusters 1, 4, 5, and 6 were identified as mesothelial cells on the basis of their expression of signature genes. *Fabp4* was widely expressed in mesothelial cells and Pafs (Figure 2D). *Pdpn*, *Pdgfra*, *S100a4*, *Fap*, *Cd34*, and *Acta2* are known as PSC markers (14, 25, 26). Our results revealed that *Pdpn* and *Pdgfra* were expressed in almost all PSCs, similar to the results of other recently published single-cell studies on PSCs. However, *S100a4*, *Cd34*, *Fap,* and *Acta2* were differentially expressed in the PSC subsets. *Sca1* and *Dpt*, which are common markers for fibroblasts based on single-cell analysis, were expressed in Clusters 0, 2, 3, 9, and 10. *Msln* and *Upk1b*, common markers of mesothelial cells, were expressed in Clusters 1, 4, 5, and 6. These results indicated that the isolated qPSCs were roughly divided into Pafs and mesothelial cells. Mesothelial cells are known to differentiate into hepatic PSCs (27), which suggests that these cell types may have densities similar to those of Pafs. Because PSCs were isolated by a density gradient based on Apte’s method, mesothelial cells with the same density as PSCs could be contaminated. Immunofluorescence confirmed that a few isolated PSCs were positive for M6A, a marker of mesothelial cells, and the remaining cells were positive for SCA1, a marker of Pafs, in nondiabetic qPSCs (Figure 2E).

### Pafs were further classified into six clusters, including the tapPaf population.

**Figure 3:**
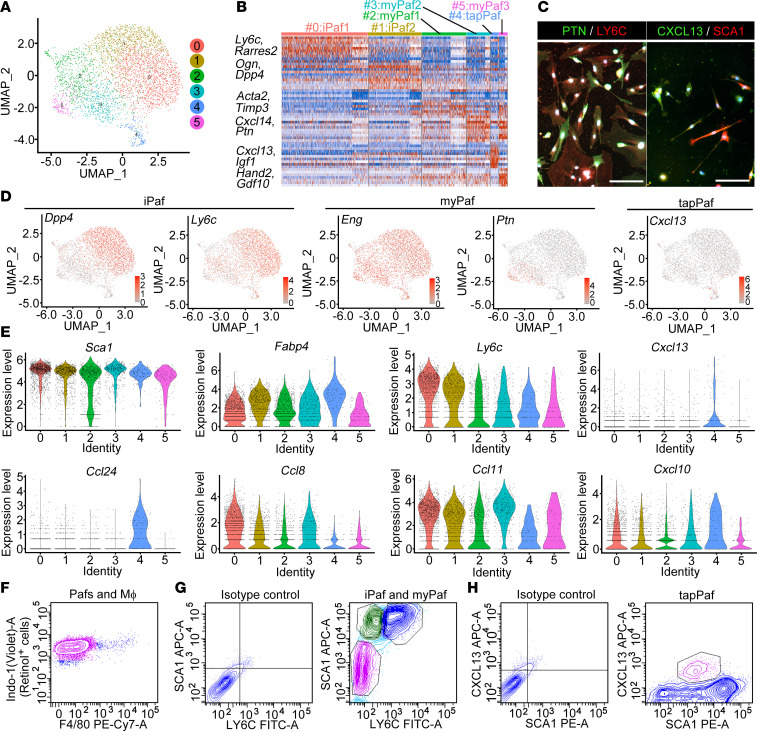
Characterization of the novel Paf subpopulation. (**A**) A separate clustering analysis further classified Pafs into six populations. (**B**) Heatmap of the Paf genes classified according to the gene expression patterns of the corresponding CAFs. (**C**) Representative immunofluorescence image showing the expression of PTN or LY6C and CXCL13 or SCA1 without copositive cells among isolated Pafs (original magnification, ×20). (**D**) UMAP image showing specific cell clusters expressing *Dpp4*, *Ly6c*, *Eng*, *Ptn*, and *Cxcl13*. (**E**) Violin blot showing the distribution of *Sca1*, *Fabp4*, *Ly6c,* *Cxcl13,* *Ccl24*, *Ccl8*, *Ccl11,* and *Cxcl10* expression. (**F**) Representative flow cytometry plot showing the sorting of specific cell populations, such as the retinol-positive and F4/80-negative populations, to exclude macrophages. (**G**) Representative flow cytometry plots for the intensities of SCA1 and LY6C cells were used to differentiate iPafs and myPafs. (**H**) Representative flow cytometry plot of CXCL13 expression in sorted tapPafs. Pafs, pancreatic fibroblasts; iPafs, inflammatory Pafs; myPafs, myofibroblastic Pafs; tapPafs, tumor immunity- and angiogenesis-promoting Pafs; Mϕ, macrophage. Scale bar:50 μm.

To examine the Paf population in detail, we further performed a separate clustering analysis in which Pafs were classified into six populations (Figure 3, A and B), i.e., populations 0 and 1, 2, 3, 5, and 4, and further categorized into three major groups. According to previous reports, the 0 and 1 populations are characterized by the gene expression of *Ly6c*, *Pi16*, and *Scara3*, which correspond to C3- and FB2-positive Pafs (Figure 3B) (5). Because these populations have a lineage similar to that of iCAFs, populations 0 and 1 were named iPafs. iPafs were subdivided into two groups, namely, iPaf1-expressing *Ly6c* (population 0) and iPaf2-expressing *Dpp4* (population 1). Populations 2, 3, and 5 were characterized by the expression of *Cxcl14*, *Ptn*, and *Cygb*, corresponding to C4-, FB1-, and *Cd105*-positive Pafs, respectively (5). For the same reason, populations 2, 3, and 5 are named myPafs. myPafs were subdivided into myPaf1-expressing *Acta2* (population 2), myPaf2-expressing *Cxcl14* (population 3) and myPaf3-expressing *Hand2* and *Gdf10* (population 5) populations. Notably, we detected a small population of Pafs corresponding to Cluster 4, characterized by the expression of *Cxcl13* and *Igf1*. This small population was named tumor immunity- and angiogenesis-promoting Pafs (tapPafs). Immunofluorescence confirmed that the cultured Pafs contained subpopulations such as Pafs expressing PTN or LY6C and those expressing CXCL13 or SCA1 (Figure 3C). There were a small number of CXCL13- and SCA1-copositive cells. Regarding the iPaf marker, the expression of *Dpp4* was restricted to the iPaf population, whereas *Ly6c* was expressed in the iPaf population and a part of the myPaf population (Figure 3D). Among the myPaf markers, *Eng* was globally expressed in Pafs, whereas *Ptn* showed limited expression in the myPaf3 population. In contrast, the expression of *Cxcl13* was restricted to the tapPaf population. RNA velocity analysis was performed to analyze the cell lineage in the Paf population. Each Paf population appeared to differentiate independently (Supplemental Figure 1; supplemental material available online with this article; https://doi.org/10.1172/jci.insight.187424DS1). Violin plot analysis confirmed that *Sca1* and *Fabp4* were expressed in all Paf subclasses (Figure 3E). *Ly6c* was an abundantly expressed gene in population 0. *Cxcl13* and *Ccl24* were exclusively expressed in population 4. The mRNA expression of cytokines and chemokines in tapPafs was characterized by low expression of *Ccl8* and *Ccl11* and the highest expression of *Cxcl10*, which is involved in the recruitment and regulation of immune cells. A modified, highly pure isolation method involving fluorescence-activated cell sorting (FACS) was adopted to sort these cell populations, in which indo-1(violet)-A and F4/80 were used to sort the retinol-positive population and exclude macrophages (Figure 3F) (28). For FACS, antibodies against SCA1, a Paf marker, LY6C, an iPaf marker (Figure 3G), and CXCL13, a tapPaf marker (Figure 3H), were used to sort each population. We successfully sorted iPafs and myPafs using both the SCA1 antibody to exclude mesothelial cells and the LY6C antibody to separate iPafs and myPafs. tapPafs were successfully sorted with SCA1 and CXCL13 antibodies. Gene Ontology analysis of tapPafs revealed strong enrichment of genes related to adipocyte differentiation and vascular development and downregulation of genes involved in the cell cycle (Supplemental Figure 2A). Pathway enrichment analysis suggested that the PI3K/AKT, TNF, NOD-like receptor, IL17, JAK-STAT, P53, and AGE-RAGE signaling pathways, which are known for PSC activation, were enriched in tapPafs (Supplemental Figure 2B).

### T2D changed the composition of Paf clusters defined by scRNA-seq.

**Figure 4:**
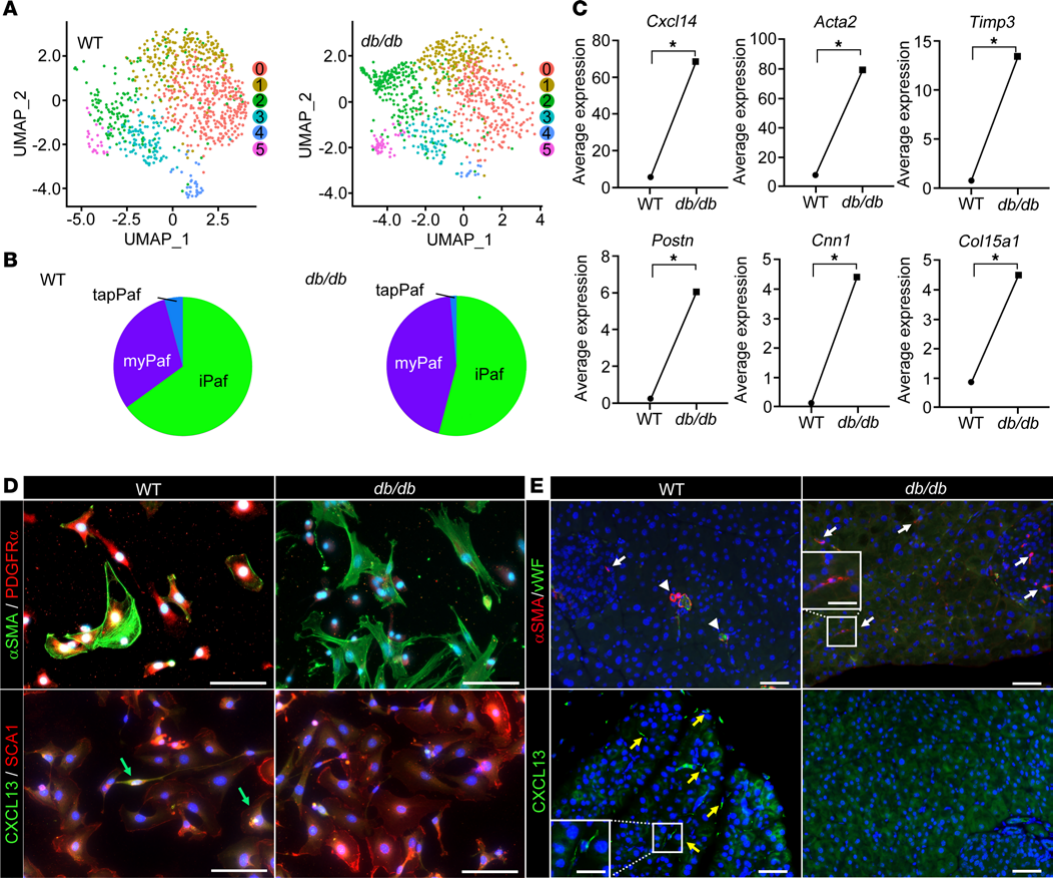
Impact of T2D on Paf subpopulations. (**A**) UMAP-visualized subpopulations of Pafs based on scRNA-seq transcriptomes pooled from WT (*n* = 2) and *db/db* mice (*n* = 2). (**B**) Pie chart showing the prevalence of different Paf cell types in WT and *db/db* mice. (**C**) Comparison of gene expression in myPafs between WT and *db/db* mice. (**D**) Representative immunocytofluorescence images showing the coexpression of α-SMA and PDGFRα or CXCL13 and SCA1 (green arrows) in Pafs from WT (*n* = 8) and *db/db* mice (*n* = 8) (original magnification, ×20). (**E**) Representative immunofluorescence images showing α-SMA–positive and vWF-negative Pafs (white arrow), α-SMA– and vWF-positive vessels (arrowheads), and CXCL13-positive Pafs (yellow arrows) in samples from WT and *db/db* mice (original magnification, ×20 and ×40 [insets]). Statistical analysis was performed by Mann-Whitney *U* test. Pafs, pancreatic fibroblasts; iPafs, inflammatory Pafs; myPafs, myofibroblastic Pafs; tapPafs, tumor immunity- and angiogenesis-promoting Pafs. ^*^P < 0.01. Scale bars: 50 μm (**D** and **E**) and 25 μm (**E**, insets).

We analyzed the changes in the populations of Pafs isolated from the pancreas of 8-week-old *db/db* mice. Compared with control mice, *db/db* mice had significantly greater body weights, fasting and fed blood glucose levels, HbA1c levels and fed insulin levels (*P* < 0.01) (Supplemental Table 1). These results confirmed that *db/db* mice constitute a model of T2D with obesity and hyperinsulinemia. Pafs isolated from *db/db* mice were classified into five populations similar to those from WT mice (populations 0–5) (Figure 4A). The myPaf population increased and the iPaf and tapPaf populations decreased in *db/db* mice according to UMAP analysis (25% versus 42%, 71% versus 57%, and 4% versus 1%, respectively) (Figure 4B). Gene expression analysis of myPafs revealed that myCAF markers such as *Cxcl14*, *Acta2*, *Timp3*, *Postn*, *Cnn1*, and *Col15a1* were significantly upregulated in *db/db* mice (*P* < 0.001) (Figure 4C). α-SMA-positive cells were rarely identified in Pafs from WT mice, while α-SMA-positive cells were dominant in Pafs from *db/db* mice (Figure 4D). In contrast, CXCL13-positive stromal cells were scattered in the pancreas of WT mice, whereas almost no CXCL13-positive cells were observed in that of *db/db* mice. In the pancreatic tissue from the WT mice, few PSCs, which were negative for vWF, were positive for α-SMA, while α-SMA-positive PSCs were identified in the pancreatic tissue from the *db/db* mice (Figure 4E). CXCL13-positive PSCs were observed in the pancreas of WT mice, whereas CXCL13-positive PSCs were rarely observed in the pancreas of *db/db* mice. Gene Ontology analysis of myPafs revealed strong enrichment of genes related to positive regulation of the cell cycle and wound healing (Supplemental Figure 3A). On the other hand, the enrichment of cytokines and chemokine-mediated signaling pathways, mainly via INF-β, was decreased. Pathway enrichment analysis of the myPafs of *db/db* mice suggested that the PI3K/AKT, MAPK, RAP1, and AGE-RAGE signaling pathways, which are known pathways involved in PSC activation, were upregulated (Supplemental Figure 3B). Collectively, these findings reveal that the myPaf population was increased and that the tapPaf population was almost eliminated in the T2D state. Gene expression analysis revealed that the myPaf population in *db/db* mice had a myCAF status with a reduction in the tapPaf population in the precancerous stage.

### tapPafs promoted lymphocyte infiltration and angiogenesis in allogenic transplanted PDAC tumors.

**Figure 5:**
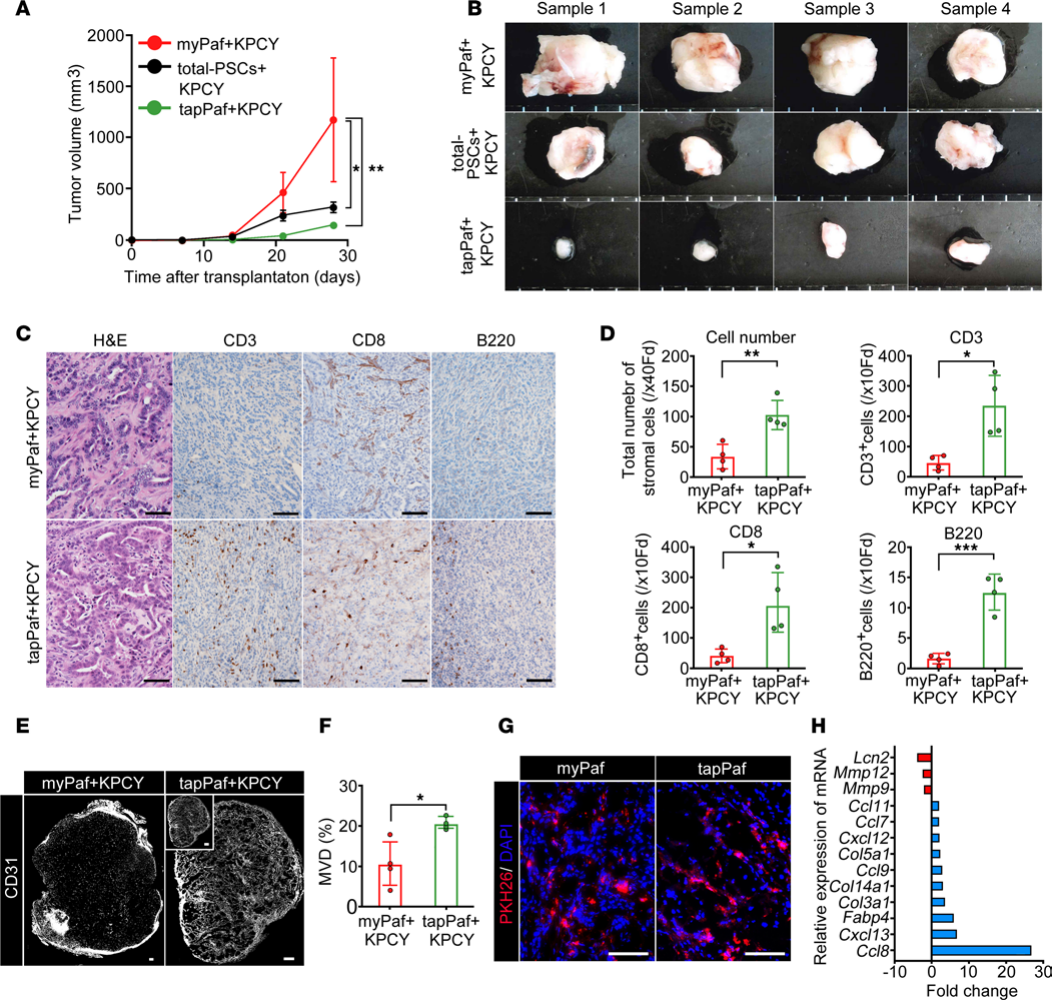
Tumor immunity and angiogenesis elicited by tapPafs in the allogeneic transplantation model mice with normal immunity. (**A**) The tumor growth rate was evaluated after the subcutaneous injection of 1.0×10^5^ KPCY tumor cells cotransplanted with 1.0×10^5^ myPafs or 2.0×10^5^ KPCY tumor cells cotransplanted with 1.0×10^5^ tapPafs or 1.0×10^5^ total PSCs in syngeneic C57BL/6J mice (*n* = 4 mice per condition). (**B**) Representative photographs showing dissected transplanted PDAC tumors from C57BL/6J mice. (**C**) Representative images of pathological H&E staining and immunohistochemical analysis of CD3-, CD8- and B220-positive immune cell infiltration in tumors transplanted with myPafs and tapPafs (original magnification, ×20). (**D**) The densities of total cells and CD3-, CD8- and B220-positive cells quantitatively evaluated in immunostained sections of the transplanted tumors (*n* = 4 per each group). (**E**) Immunofluorescence for CD31 revealing microvessel channels in the tumors. The full size of the tumor is shown in the inset (original magnification, ×20). (**F**) MVD quantified in the CD31-positive area using Otsu’s method for image thresholding (*n* = 4 per each group). (**G**) PKH26-labeled Pafs (red fluorescence) retained in tumors 4 weeks after transplantation (original magnification, ×20). (**H**) Comparison of mRNA fold changes measured by microarray. Statistical analysis was performed by 2-way ANOVA with post hoc multiple-comparison tests. MVD, microvascular density; Pafs, pancreatic fibroblasts; myPafs, myofibroblastic Pafs; PSCs, pancreatic stellate cells; tapPafs, tumor immunity- and angiogenesis-promoting Pafs; Fd, field. The data are presented as the means ± SDs. ^*^P < 0.05, ^**^ *P* < 0.01, ^***^ *P* < 0.001. Scale bars: 100 μm (**C** and **G**) and 300 μm (**E**).

Concurrent subcutaneous transplantation of PDAC cells and tapPafs was conducted to evaluate angiogenesis and the response of PDAC cells in recipients with full immunity. To analyze the antitumor immunity of tapPafs, a mixture of Pafs sorted by FACS and a KPCY cell line generated from C57BL/6J mice with PDAC were subcutaneously transplanted into 10-week-old C57BL/6J mice with full immunity via allogenic transplantation. Mice transplanted with 1.0 × 10^5^ total-PSCs, which are crude PSCs isolated from the pancreas by Apte’s method, + 1.0 × 10^5^ KPCY cells or 1.0 × 10^5^ myPafs + 1.0 × 10^5^ KPCY cells. The transplanted cells formed subcutaneous tumors during a 4 weeks of observation. (Figure 5, A and B). In contrast, because tumor formation was not observed with 1.0 × 10^5^ tapPafs + 1.0 × 10^5^ KPCY cells, a doubled number of KPCY cells (2.0 × 10^5^) were transplanted with tapPafs, resulting in the formation of tumors. Dissected KPCY tumors transplanted with myPafs were significantly larger and showed invasive growth after four weeks (Figure 5B). The density of stromal cells in KPCY tumors with tapPafs was significantly greater than that in KPCY tumors with myPafs in H&E-stained sections (Figure 5, C and D). In support of the increase in stromal cell density, KPCY tumors transplanted with tapPafs had more intrastromal immune cells than those transplanted with myPafs (Figure 5, C and D). In the tumors transplanted with tapPafs, CD3-positive and CD8-positive T cells and B220-positive B cells abundantly infiltrated the tumors. The microvascular density (MVD) of tumors transplanted with tapPafs was significantly greater than that of tumors transplanted with myPafs, as visualized by whole-tumor immunofluorescence for CD31 (Figure 5, E and F). PKH26 staining confirmed that the transplanted Pafs survived in the engrafted tumor 4 weeks after transplantation (Figure 5G). Microarray analysis of tumors generated by concurrent subcutaneous transplantation of KPCY cells and tapPafs showed that *Cxcl13*, a marker of tapPafs and *Fabp4*, a marker of all types of Pafs, were elevated in tumors transplanted with tapPafs compared with those transplanted with myPafs (Figure 5H). Similarly, the expression of *Ccl7*, *Ccl8*, *Ccl9*, and *Ccl11*, which are chemokines involved in immunity, was increased in tumors transplanted with tapPafs. Volcano plot analysis revealed a significant increase in *Muc1*, *Muc4*, *MMPs*, *IL1a*, *Tnfrsf11b*, *Serpine1,* and *Tmc5* expression levels in tumors transplanted with myPafs, which are known to be elevated with malignancy (Supplemental Figure 4A). In tumors transplanted with tapPafs, *Cxcl13*, *Fabp4*, *S100a4,* and *Cd34*, markers of PSCs, and *Dpt*, a marker of fibroblasts, were significantly elevated. These results suggest that tapPafs retained their own traits as a PSC population within PDAC tumors. Gene set enrichment analysis revealed activation of the immune response, lymphocyte chemotaxis, regulation of vascular angiogenesis, and development in tumors transplanted with tapPafs (Supplemental Figure 4B). These data suggest that tapPafs could activate tumor immunity, including B- and T-cell immunity, resulting in the inhibition of PDAC progression.

### Diet-induced obesity elicited a reduction in the tapPaf population and an increase in the myPaf population in transplanted PDAC tumors.

**Figure 6:**
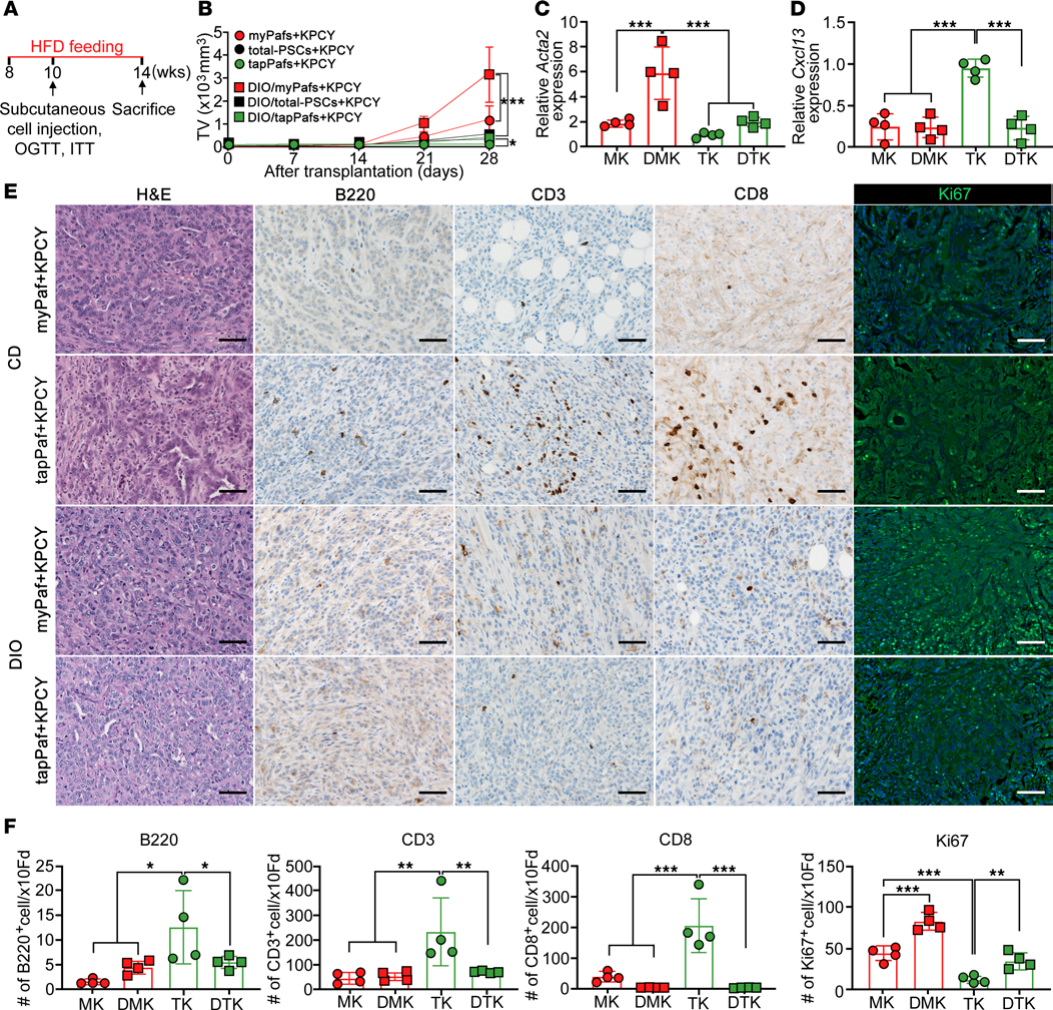
Effects of DIO on tumor immunity in allogeneic transplantation–model mice with normal immunity. (**A**) Schematic representation showing the protocol for the subcutaneous injection of 1.0 × 10^5^ KPCY tumor cells cotransformed with 1.0 × 10^5^ myPafs, tapPafs, or total PSCs in syngeneic B6 mice fed a high-fat diet to generate DIO. (**B**) The tumor growth rate was evaluated in DIO B6 mice after subcutaneous tumor transplantation, with *n* = 4 mice per condition. (**C**) *Acta2* and (**D**) *Cxcl13* mRNA expression was quantitatively evaluated in the tumors (*n* = 4 per each group). (**E**) Representative images of pathological H&E staining and IHC analysis of immune cell infiltration of CD3, CD8, and B220 in tumors transplanted with myPafs and tapPafs (original magnification, ×20). (**F**) The densities of total cells and CD3-, CD8- and B220-positive cells quantitatively evaluated in IHC sections of the transplanted tumors (*n* = 4 per each group). The data are presented as the mean ± SD. Statistical analysis was performed by 2-way ANOVA with post hoc multiple-comparison tests. TV, tumor volume; HFD, high-fat diet; OGTT, oral glucose tolerance test; ITT, insulin tolerance test; DIO, diet-induced obesity; CD, control diet; Pafs, pancreatic fibroblasts; myPafs, myofibroblastic Pafs; tapPafs, tumor immunity- and angiogenesis-promoting Pafs; PSCs, pancreatic stellate cells: MK, mPafs+KPCY; DMK, DIO/mPafs+KPCY; TK, tapPafs+KPCY; DTK, DIO/tapPafs+KPCY; Fd, field. ^*^P < 0.05, ^**^P < 0.01, ^***^P < 0.001. Scale bar: 100 μm.

To analyze the effects of insulin resistance, such as that in T2D, on Pafs in vivo, a diet-induced obesity (DIO) model was generated. After two weeks of high-fat diet (60% of calories from fat) (HFD) administration to C57BL/6J mice, subcutaneous allogeneic implantation of KPCY cells with Pafs was performed, and the mice were observed for 4 weeks (Figure 6A). The DIO mice showed significant glucose and insulin intolerance after the 2 g/kg glucose oral glucose tolerance test compared with animals in the control chow diet (10% total calories from fat) (CD) group (Supplemental Figure 5, A and B). In the subcutaneously transplanted DIO mice, tumor formation was observed in all the mice in the group transplanted with 1.0 × 10^5^ KPCY cells and 1.0 × 10^5^ tapPafs, 1.0 × 10^5^ KPCY cells and 1.0 × 10^5^ total-PSCs, or 1.0 × 10^5^ KPCY cells and 1.0 × 10^5^ myPafs (Figure 6B). The tumor growth rate was significantly greater in all the groups fed a HFD than in the CD groups. In particular, the tumor growth rate of the myPaf + KPCY tumors was the highest in the DIO model, whereas that of the tapPaf + KPCY tumors was comparable to that of the myPaf + KPCY tumors in CD mice. DIO significantly increased *Acta2* expression in the tumors transplanted with myPafs but slightly increased *Acta2* expression in the tumors transplanted with tapPafs (Figure 6C). *Cxcl13* expression was comparable between CD and DIO mice transplanted with myPafs and KPCY cells, whereas *Cxcl13* expression was significantly lower in the DIO mice transplanted with tapPafs and KPCY cells than in the CD mice (Figure 6D). According to the IHC results, the infiltration of CD45-, CD3-, CD8- and B220-positive cells was comparable in the myPafs + KPCY tumors regardless of DIO (Figure 6, E and F). The number of all kinds of positive cells was significantly greater in tapPafs + KPCY tumors than in myPafs + KPCY tumors, whereas the number of positive cells was significantly decreased in tapPafs + KPCY tumors of the DIO mice. The Ki67 index, which reflects cell proliferation ability, was significantly increased by DIO in myPafs + KPCY tumors. The Ki67 index was significantly lower in tapPafs + KPCY tumors than in myPafs + KPCY tumors but was significantly increased by DIO. These results suggest that DIO enhances the CAF status of myPafs and decreases the immunocompetence of tapPafs in transplanted PDAC tumors.

### An increase in activated PSCs and a decrease in CXCL13-positive PSCs are associated with PDAC accompanied by T2D in humans.

**Figure 7:**
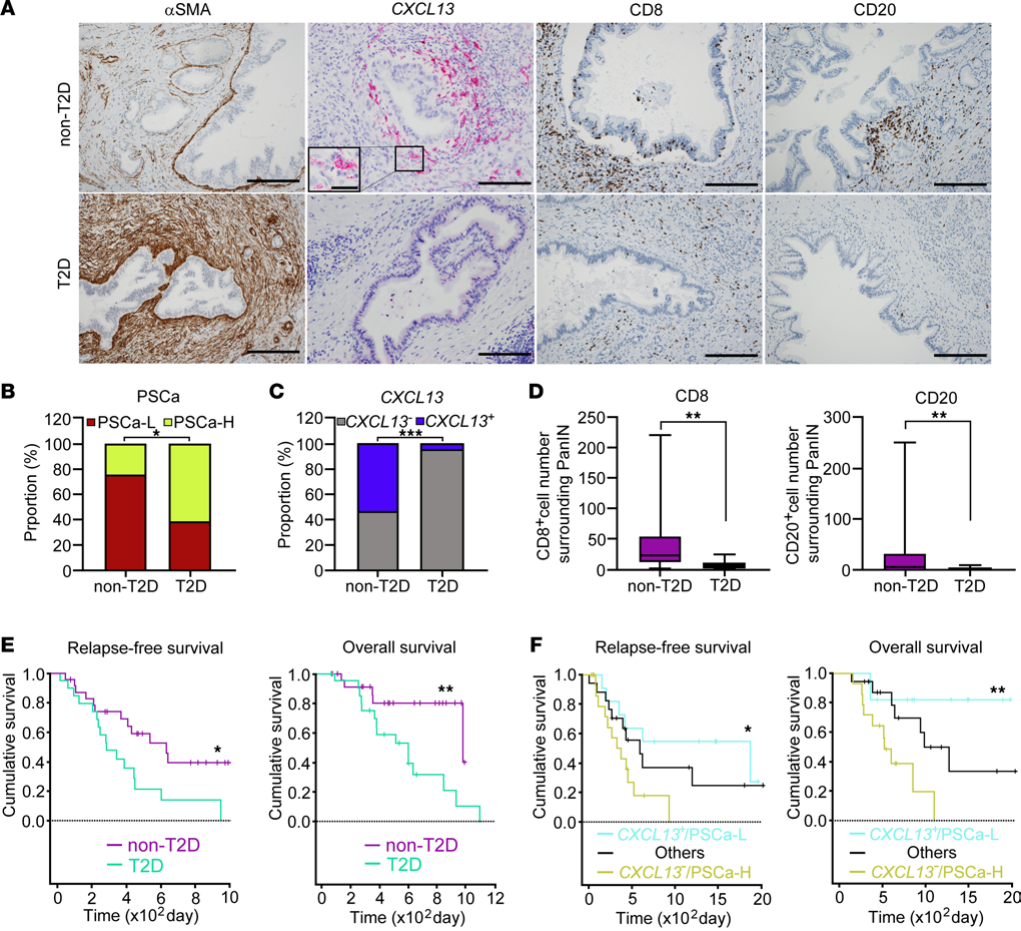
PSC activation score and *CXCL13* expression around PanIN lesions in human PDAC samples. (**A**) Representative images of IHC and in situ hybridization illustrating the expression of α-SMA, *CXCL13*, CD8, and CD20 surrounding PanIN lesions (original magnification, ×20 and ×40 (inset)). (**B**) PSC activation was evaluated by the PSCa score in individuals who did not have T2D (*n* = 24) and who did have T2D (*n* = 21). (**C**) The presence of *CXCL13*-positive stromal cells surrounding PanIN lesions evaluated in sections via in situ hybridization in individuals who did not have T2D (*n* = 24) and who did have T2D (*n* = 21). (**D**) The density of CD8- and CD20-positive cells surrounding PanIN lesions quantitatively evaluated in immunostained sections according to the presence of T2D and differences in the PSCa score (*n* = 24: non-T2D; and *n* = 21: T2D). Kaplan-Meier analysis was performed according to the presence of T2D and differences in *CXCL13*-positive stromal cells and PSCa to compare RFS and OS (**E** and **F**) in PDAC samples. The data are presented as the means ± SDs. For **D**, box and whiskers are median and 25% interquartile intervals. Statistical analysis was performed by Fisher’s exact test and Mann-Whitney *U* test. For multiple comparisons, the Z test with Bonferroni adjustment was used. Survival curves were calculated with Kaplan–Meier analysis. PanIN, pancreatic intraepithelial neoplasia; T2D, type 2 diabetes; PSCs; pancreatic stellate cells; PSCa, pancreatic stellate cell activation; PSCa-L, PSCa-low; PSCa-H, PSCa-high; PDAC, pancreatic ductal adenocarcinoma. ^*^ *P* < 0.05, ^**^ *P* < 0.01, ^***^ *P* < 0.001. Scale bar: 100 μm and 25 μm (inset).

To investigate the effects of PSCs, including tapPafs, on PDAC, we analyzed the PSC status and immune cell infiltration around areas of pancreatic intraepithelial neoplasia (PanIN) in patients with T2D. The clinical characteristics of the individuals with PDAC who do not have T2D and patients with T2D are shown in the Supplemental Table 2. In human pancreatic cancer samples with PanIN, individuals who did not have T2D presented sparse α-SMA-positive areas around the PanIN, whereas individuals with T2D presented α-SMA-positive areas surrounding the PanIN (Figure 7A). T2D patients had significantly higher PSC activation (PSCa) scores than individuals who did not have T2D did, as evaluated by the intensity of α-SMA staining (Figure 7, A and B). Although not numerous, the presence of *CXCL13*-positive stromal cells surrounding the PanIN in individuals who did not have T2D was revealed by in situ hybridization, while the number of *CXCL13*-positive stromal cells was significantly decreased in patients with T2D (Figure 7, A and C). The group with *CXCL13*-positive stromal cells had significantly lower HbA1c levels and significantly shorter duration of diabetes compared with CXCL12-negative stromal cells (Supplemental Figure 6). The infiltration of CD8- and CD20-positive cells was significantly lower in individuals with T2D than in individuals who did not have T2D (Figure 7D). Regardless of the presence of T2D, the individuals with high PSCa scores showed significantly decreased infiltration of CD8-positive cells and CD20-positive surrounding the PanIN compared with the people with low PSCa scores (Supplemental Figure 7A). Compared with those without *CXCL13*-positive stromal cells, those with *CXCL13*-positive stromal cells had significantly more CD8- and CD20-positive lymphocytes surrounding the PanIN (Supplemental Figure 7B). We further evaluated intratumoral tertiary lymphoid structures (TLSs). Intratumoral TLSs were defined as lymphoid cell aggregates in which CD20-positive B cells were concentrated in the center and CD8-positive T cells were located in the periphery (Supplemental Figure 8A). The density of TLSs was significantly lower in individuals with T2D than in those without T2D (Supplemental Figure 8B). Patients with a lack of *CXCL13*-positive stromal cells and high PSCa scores had a significant lower density of TLSs than patients with *CXCL13*-positive stromal cells and low PSCa scores (Supplemental Figure 8C). The Kaplan-Meier survival curve clearly indicated shorter relapse-free survival (RFS) and overall survival (OS) in patients with T2D than in those without T2D (Figure 7E). RFS and OS were also significantly shorter in patients with an absence of *CXCL13*-positive stromal cells and high PSCa scores (PSCa-high/*CXCL13*^–^) than in patients with *CXCL13*-positive stromal cells and low PSCa scores (PSCa-low/*CXCL13*^+^) (Figure 7F). Univariate analysis of RFS and OS revealed that T2D was a significant risk factor for shorter survival, whereas PSCa-low/*CXCL13*^+^ was a factor significantly improving prognosis. (Supplemental Table 3). PSCa-low/*CXCL13*^+^ was an independent factor for RFS according to multivariate analysis (Supplemental Table 4). Therefore, our results revealed the activation of PSCs and a reduction in the number of *CXCL13*-positive stromal cells, which may be associated with a worse prognosis in human patients with PDAC and T2D.

## Discussion

Currently, PSCs are classified into two or three groups according to mRNA expression. Hutton et al. reported that CD105 is a common marker for classifying PSCs and CAFs (6). Dominguez et al. reported that PSCs can be classified into two groups: C3- and *Cd105-*expressing C4-PSCs (6, 7). Hosein et al. reported that PSCs can be classified into three groups, FB1, FB2, and FB3, without mentioning *Cd105* expression (5). Our study revealed that *Cd105* could be used to distinguish mesothelial cells from Pafs because the *Cd105*-negative cell population expresses mesothelial cell markers. The classifications in the previous reports are not identical to our results but correlate to some extent. Interpreting the current results on the basis of previous reports, C3 and FB2 are assumed to correspond to iPafs; C4 and FB1 correspond to myPafs; and FB3- and CD105-negative Pafs correspond to mesothelial cells. Furthermore, a small proportion of Paf population expressing *Cxcl13*, named tapPafs, was identified in our study. In this study, isolated PSCs were maintained without passaging, long-term culture, or the use of activating agents to preserve a quiescent state, which could lead to the identification of tapPafs as a small subpopulation of qPSCs.

CXCL13 is associated with the recruitment of B-cell and T-cell subsets (29, 30). A cancer microenvironment enriched with CXCL13 elicits the recruitment of CXCR5-expressing leukocytes (31). Although tapPafs express *Cxcl13*, whether it is functional or a mere marker of tapPafs is unclear. In this study, marked infiltration of lymphocytes was demonstrated in tumors formed by concurrent subcutaneous transplantation of PDAC cells and tapPafs in mice with full immunity. Furthermore, the presence of *CXCL13*-positive stromal cells and low PSCa surrounding PanIN cells were proportionally correlated with the density of TLSs in patients with PDAC. These findings indicate that CXCL13 secreted from tapPafs can activate the intratumoral immune system, particularly B-cell immunity. TLSs are ectopically formed aggregates of lymphoid cells for which CXCL13 is a chemoattractant that promotes lymphocyte recruitment (32, 33). The majority of patients with “immune-rich” PDAC have TLSs and exhibit the best outcomes (34, 35). Delvecchio et al. reported that intratumoral injection of CXCL13/CCL21 enhanced immune checkpoint inhibitor (ICI) efficacy via TLS formation in an orthotopic PDAC-transplanted mouse model (36). Several compounds, such as all-trans retinoic acid, vitamin D, calcipotriol, and N-acetyl cysteine, are thought to suppress or remodel CAFs (37–41). Therefore, the administration of these compounds may increase the tapPaf population and promote both the infiltration of immunocompetent cells and the formation of TLSs. Restoration of the tapPaf population may be a novel option for ICI therapy in patients with PDAC complicated with T2D.

PDAC is characterized by a dense stroma with limited vascularization, leading to impaired drug delivery, poor immune cell infiltration, and a hypoxic and acidic tumor microenvironment (42–45). In contrast, simply removing stromal tissue does not constitute radical treatment of PDAC (46, 47). Vascular normalization aimed at improving drug delivery could be a good strategy for this type of carcinoma (48, 49). A high density of vascular channels in tumors is known to increase the number of immune cells, which can lead to a better prognosis (50, 51). Gene Ontology analysis revealed that tapPafs significantly increased vascular formation. Furthermore, concurrent subcutaneous transplantation of PDAC cells and tapPafs resulted in a greater MVD than did transplantation with myPafs. These findings suggest that tapPafs are expected to overcome the dense stromal wall in PDAC via an increase in vascular density.

T2D is known to be an oncogenic and poor prognostic factor for patients with PDAC (18–21). We previously reported the involvement of epigenetic changes such as promoter methylation and miRNA expression in the poor prognosis of patients with PDAC complicated with long-term T2D (19–21). However, the mechanism mediated by PSCs has not been fully elucidated in patients with PDAC complicated with T2D. In a previous study, we demonstrated that long-term T2D activated PSCs via RAGE signaling and TGF-β expression, particularly in the context of dyslipidemia (19). These results suggest that PSCs had acquired the properties of cancer-promoting CAFs even before the onset of cancer in patients with T2D. We revealed that T2D increased the density of α-SMA-positive activated PSCs in the pancreas. T2D further decreased the iPaf population and increased the myPaf population, simulating the CAF status in PDAC. Ogawa et al. classified the PDAC stroma into three types: collagen-rich stroma, fibroblast activation protein α(FAP)-dominant fibroblast-rich stroma, and α-SMA-dominant fibroblast-rich stroma (9). Among these patients, those with α-SMA-dominant fibroblast-rich stroma exhibited poorer prognoses. Thus, an increase in the population of myPafs expressing α-SMA before the onset of PDAC is a factor associated with a worse prognosis in patients with PDAC complicated with T2D in combination with epigenetic changes in PDAC cells.

Biffi et al. reported that mouse PSCs transdifferentiate into either myCAFs or iCAFs via different pathways (8). Furthermore, Mizutani et al. reported that the proportion of cancer-restraining CAFs expressing Meflin was reduced in patients with PDAC (11). Although the specific factors eliciting these transitions have not been identified, previous research has shown that aging, hypoxia, and TGFβ signaling decrease Meflin expression (52). In our previous study, diabetic status increased the expression of RAGE in stromal tissues in patients with PDAC, which may increase the expression of TGFβ (19). Our data revealed that each Paf population appeared to differentiate independently and that the proliferation of each Paf population might be affected by T2D. Thus, T2D could serve as a new clinical modulator for altering the number and proliferation of Paf subpopulations in the precancerous stage (53, 54).

Previous reports have shown that myPafs can increase the malignant potential of cancer cells in a TGFβ-dependent manner (8, 19). Gene Ontology analysis revealed upregulated pathway-related cell proliferation via PI3K in myPafs. In support of these results, PDAC cells transplanted with myPafs had a greater Ki67 index for tumor cells than those transplanted with tapPafs. Although the mechanism underlying the increase in Ki67-positive PDAC cells in this study is not fully understood, our results confirm that tumor progression is complicated by stromal components in PDAC. Furthermore, T2D is a clinical factor that can increase the proliferation of PDAC cells by changing the proportions of PSC subtypes. Strict management of T2D is expected to drive PSCs back to an appropriate ratio, resulting in a decrease in cell proliferation in PDAC.

Our study has several limitations. First, tapPafs were isolated using flow cytometry in this study. Therefore, regarding the properties of tapPafs, the results obtained from scRNA-seq analysis may differ from those obtained from isolated and cultured cells because of possible cell activation by FACS. Second, it is unclear which types of immune cells are most strongly affected by tapPafs and how they exert their antitumor effects on the basis of KPCY cell transplantation experiments in C57BL/6J mice, in which most immune cells are functional. Third, *CXCL13*-knockdown experiments were not performed in the present study. Knockdown of *CXCL13* in tapPafs could reveal a direct role and potential therapeutic application of CXCL13 in PDAC progression. Fourth, we evaluated T2D models (*db/db* and DIO models), which are hyperinsulinemic and highly insulin resistant. These findings suggest that insulin or insulin resistance may be more involved in changes in Paf populations than in glycemic changes in patients with PDAC. More details concerning glycemic control and therapeutic content should be addressed in the future.

Despite the above shortcomings, we identified a small Paf subpopulation, tapPafs, from isolated mouse qPSCs. tapPafs could enhance tumor immunity and vasculogenesis in PDAC. T2D modified the subpopulation of Pafs with an increase in myPafs and a decrease in iPafs and tapPafs, which facilitated tumor formation. Modulation of PSC subpopulations, such as an increase in the tapPaf population, may suppress tumor formation in PDAC, which may be achieved by strict control of T2D. Further clinical trials or prospective studies are needed to support future therapeutic and clinical applications of tapPafs.

## Methods

### Sex as a biological variable.

Our study exclusively examined female mice. It is unknown whether the findings are relevant for male mice.

### Mice and cell lines.

Female WT and *db/db* mice were purchased from CLEA Japan, Inc.. All the mice were maintained under specific pathogen-free conditions. All animal experiments were performed in the animal facility of the Hirosaki University Graduate School of Medicine. The mice were allowed ad libitum access to food and water and housed in polycarbonate cages, each containing 4 mice, with wire lids and hardwood chips for bedding at a constant temperature (23 ± 2 °C) under a 12-hour light–dark cycle (lights on: 7:00 a.m.–7:00 p.m.). For the animal experiments, we adhered to ARRIVE 2.0. Thirty 8-week-old female WT mice were randomly divided into a CD group and an HFD group (Research Diets, Inc.) and maintained for 2 weeks to generate the DIO mouse model. The mouse PDAC cell line BL6KPCY- from C57/BL6J fully backcrossed KPCY mice was obtained from Kerafast, Inc. The animals used for the CD models were as follows: myPaf+KPCY model, 10-week-old C57BL/6J mice (*n* = 4); total-PSC+KPCY model, 10-week-old C57BL/6J mice (*n* = 4); and tapPaf+KPCY model, 10-week-old C57BL/6J mice (*n* = 4). The animals used for the DIO models were as follows: DIO/myPaf+KPCY model, 8-week-old C57BL/6J mice (*n* = 4); DIO/total-PSC+KPCY model, 8-week-old C57BL6 mice (*n* = 4); and DIO/tapPaf+KPCY model, 8-week-old C57BL/6J mice (*n* = 4). In the case of tumor transplantation experiments, animals that showed significant emaciation due to infection or extreme tumor growth were excluded from the experiments. The mice were euthanized with 3% isoflurane anesthesia after an overnight fast (16 hours) in the animal facility of the Hirosaki University Graduate School of Medicine. H&E staining was applied to screen histological changes of the pancreas. Silver staining was performed to evaluate the deposition of reticular fiber in the pancreas. Immunofluorescent staining was performed, as previously described, with the antibodies shown in Supplemental Table 5 (55). The pancreas and tumor tissues were fixed immediately upon harvest in 10% neutral-buffered formalin overnight and embedded in paraffin. Then, serial 4 μm–thick deparaffinized sections were immersed in 0.01 mol/L citrate buffer (pH 6.0) and subsequently placed in a pressure chamber (Pascal, Agilent Technologies Inc.) for antigen retrieval at 125°C for 10 minutes. For double immunofluorescence staining, the serial sections of each sample were incubated with the combination of primary antibodies overnight at 4°C. After being washed with TBS-T, the following secondary fluorescent antibodies were added and incubated for 2 hours at room temperature. Images were obtained with a CQ1 confocal image cytometer (Yokogawa Electric Corp.).

### Isolation of PSCs and flow cytometry.

PSCs were isolated from the healthy pancreas of C57BL/6J mice using the modified Apte method (12, 13, 19). After isolation, PSCs were seeded at 5.0 × 10^5^ in 35 mm dishes (60% confluent) in DMEM/F12/10% FCS/1% antibiotics. The medium was changed on the second and fourth days. We used primary qPSCs without passage 5 days after isolation in our experiments (26). In the case of FACS analysis, immediately after isolation and 5 minutes on ice, anti-Sca1-PE, anti-Ly6c-APC and anti-F4/80-PECy5 or anti-Sca1-FITC, anti-CXCL13-APC and anti-F4/80-PECy5 (all from BioLegend) were applied at a concentration of 0.25 mL of each antibody in 100 mL of FACS buffer per 1 × 10^6^ cells. The cells were sorted via flow cytometry (FACSAria II instrument, BD Biosciences) using the gating strategy described in the manuscript. Each PSC subtype sorted in DMEM/F12 was centrifuged and aspirated, and RNA was isolated using TRI reagent (Molecular Research Center Inc.) according to the manufacturer’s instructions. Reverse transcription and quantitative PCR for each RNA and the endogenous control β2m were conducted using TaqMan RNA Assays (Thermo Fisher Scientific). Real-time PCR measurements with specific TaqMan primers for each RNA were performed in duplicate on the ABI PRISM 7000 system (Applied Biosystems), and the mean Ct value for each sample was determined. The relative expression of each RNA was calculated using the comparative Ct method.

### Immunocytochemistry.

Isolated PSCs were maintained on chamber slides, fixed with 4% paraformaldehyde for 15 minutes at room temperature, and permeabilized with 0.4% Triton X-100 in PBS for 5 minutes. The cells were incubated with primary antibodies shown in Supplemental Table 5, overnight at 4°C. After the samples were washed with 0.4% Triton X-100 in PBS, secondary fluorescent antibodies were added and incubated for 2 hours at room temperature. Images were obtained with a CQ1 confocal image cytometer (Yokogawa Electronic Corp.).

### scRNA-seq and analysis of PSCs.

We processed PSCs pooled from two mice per group using the Chromium Single Cell 3’ GEM Library & Gel Bead Kit v3 (10×Genomics), followed by sequencing on an Illumina NovaSeq platform (Illumina Inc.). The sequencing data were mapped to the reference genome (refdata-gex-mm10-2020-A) using Cell Ranger 5.0.0, and only cells expressing between 200 and 9,000 genes and having fewer than 20% of the total number of mitochondrial gene counts were considered for downstream analysis. We normalized the gene expression data via SCtransform, applied cell cycle effect correction, standardized the data, and removed outliers. Feature extraction was based on the variability in normalized gene expression. We used velocyto to count the number of transcript reads before and after splicing. Two 8-week-old WT C57BL6 mice and two 8-week-old *db/db* mice were used for the experiments.

### Microarray analysis.

Gene expression profiling was performed using a mouse Clariom D assay (Affymetrix). Total RNA (100 ng) was subjected to microarray analysis as previously reported (26). The selected genes were then subjected to volcano plotting and hierarchical clustering.

### Enrichment analysis.

Gene Ontology and Kyoto Encyclopedia of Genes and Genomes pathway enrichment analyses were performed for the cluster-specific genes, which were defined as genes detected in more than 10% of the cells composing the cluster. Transcription factor–binding site enrichment analysis was conducted using the oPOSSUM web service (28). Pathways with adjusted *P* < 0.05 and TF motifs with z scores >10 were considered significantly enriched.

### Subcutaneous PDAC cell and PSC cotransplantation experiments.

Syngeneic 12-week-old female B6 mice were used for the subcutaneous cotransplantation studies in this study. BL6KPCY cells and PSCs were cultured in DMEM/F12. A PKH26 Cell Linker Kit (Merck KGaA) was used to label PSCs. On the day of injection, the dissociated cells were washed twice with ice-cold PBS and counted in duplicate using a TC20 Automated Cell Counter (Bio-Rad) in bright-field mode. Ice-cold growth factor-reduced Matrigel (Corning) was added via precooled pipette tips to obtain a final concentration of each cell type of 5,000 cells/μL, and the mixture was gently mixed on ice. BD Lo-Dose U-100 Insulin Syringes (Becton Dickinson) were used to accurately measure 20 mL of the cell/Matrigel mixture with no dead volume, which was injected subcutaneously into the right flank of each mouse (therefore yielding 1 × 10^5^ cancer cells and 1 × 10^5^ PSCs). Mice were excluded from the experiments if they showed extreme emaciation and a tendency toward infection. Tumor tissues were rapidly frozen and sectioned using a cryostat microtome, and PKH26 (red) fluorescence signals were captured using a fluorescence microscopy at 4 weeks after transplantation. For gene expression analysis, subcutaneous tumors were dissected on day 14 and lysed in 1 mL of TRI reagent, and total RNA was isolated using RNeasy kits (QIAGEN Sciences) following the manufacturers’ recommendations.

### Evaluation of patients with PDAC who underwent surgical resection.

Forty-five PDAC samples surgically resected from PanIN lesions in patients were obtained between 2014 and 2019 from the archive files of Hirosaki University Hospital. The clinicopathological characteristics of the participants are shown in Supplemental Table 2. Diabetic patients fulfilled the criteria for diabetes proposed by the Japan Diabetes Society (56). The pathological diagnosis of PDAC and PanIN was reevaluated according to the 2019 WHO classification of tumors of the digestive system and graded based on the UICC TNM classification of malignant tumors (8th edition) by three in-house pathologists. PanIN can be divided into three grades based on the cytoarchitectural atypia (57). The activation of PSCs surrounding areas of PanIN was evaluated according to the PSC activation score (2). To exclude the influence of the diameter of the duct with PanIN, the formula described below was applied:

PSC activation score = [maximum width of the αSMA-positive area surrounding the PanIN (μm)/major axis of the duct (μm) × minor axis of the duct (μm)] × 10^4^. Identification and quantification were both performed using multiple serial H&E-stained and IHC-stained paraffin sections, as previously described (21–23). Sections were stained with antihuman antibodies against CD8 (Dako, 1:200) and CD20 (Dako, 1:200) to validate the presence of TLSs by demonstrating the content of T cells and B cells. Three in-house pathologists, blinded to each other’s scores and patient outcomes, evaluated TLSs in whole-tissue slides immunostained with CD8 and CD20 using a semiquantitative TLS scoring model in which both lymphoid aggregates and follicles were considered TLSs. In these semiquantitative TLS scoring model, the overall number of TLSs was scored on a four-tiered scale in the tissue as follows: 0, none or one TLS; 1, sparse TLSs; 2, moderate presence of TLSs; and 3, strong and heavy presence of TLSs, equivalent to that seen in a lymph node.

In situ hybridization was performed via manual RNAscope chromogenic assays using an RNAscope 2.5 HD Reagent Kit-RED according to the manufacturer’s instructions (ACDBio). The chromogenic probes used were Hs-CXCL13 and negative control probe. Specimens from participants with PDAC were divided into a *CXCL13*^–^ group and a *CXCL13*^+^ group based on the presence of *CXCL13*-positive stromal cells surrounding PanIN lesion.

### Statistics.

The data are presented as the means ± SDs. All the statistical analyses were conducted using JMP software (version 10.0.2; SAS Institute Inc.). Continuous variables were compared with Student’s *t* test or the Mann–Whitney *U* test. Categorical variables were compared by χ^2^ analysis or the Mann–Whitney *U* test, where appropriate. Comparisons of average values between the two groups were performed via the nonparametric Mann–Whitney *U* test. For multiple comparisons, two-way ANOVA with Bonferroni adjustment was used. A simple regression was performed for the correlation analysis. RFS was defined as the time elapsed between surgical resection and tumor recurrence. OS was calculated as the time between surgery and death from any cause. Survival curves were generated via Kaplan–Meier analysis, and *P* values were determined by the log rank test for censored survival data. All tests were 2 tailed, and a *P* value of < 0.05 was considered statistically significant.

### Study approval.

All animal experiments were performed according to a protocol that was approved by the Animal Care and Use Committee of the Hirosaki University School of Medicine (#M21018). This study was approved by the ethical committee of the Hirosaki University Graduate School of Medicine (#2023-155), and signed written consent forms were obtained from all participants to use the surgical specimens and recordings during their treatments. This study was performed in accordance with the Declaration of Helsinki.

### Data availability.

All supporting data for this study are provided within the main text and Supplemental Materials. Individual data points from graphs in both the main text and supplemental materials are available in the Supporting Data Values file. Additional raw data can be requested from the corresponding author. Raw data related to human participants will be provided after deidentification and in compliance with applicable privacy laws and data protection regulations. Microarray data are available at NCBI under GEO accession number GSE262456. scRNA-seq data are available at NCBI BioProject under accession code PRJDB17785. A Supporting Data Values file is provided as supplemental material.

## Author contributions

YH was the lead for conceptualization, data curation, formal analysis, investigation, methodology, and writing of the original draft. HM was the lead for conceptualization, funding acquisition, investigation, project administration, supervision, and reviewing and editing the manuscript.

TY was supporting in formal analysis, investigation, acquisition of resources, and visualization. SS was supporting in data curation, formal analysis, and methodology. KY, TS, ZW, HK, MR, SO, HT, and AI were supporting in data curation and formal analysis. NK was supporting in formal analysis and acquisition of resources. KI was supporting in acquisition of resources. SU and KH were supporting in supervision and reviewing and editing the manuscript.

## Supplementary Material

Supplemental data

Supporting data values

## Version 1 06/23/2025

Electronic publication
