# Supplementary material for: Type 2 diabetes alters quiescent pancreatic stellate cells to tumor-prone state
Source: JCI Insight. 2025 Jun 23;10(12):e187424. doi: 10.1172/jci.insight.187424 (PMC12220947; doi:10.1172/jci.insight.187424)
Supplement: Supplemental data [file jciinsight-10-187424-s206.pdf]

Supplementary Table S1. Clinical characteristics of *db/db* mice

	Wild type	*db/db*
Body weight (g)	24.7 ± 0.9	39.2 ± 2.6***
HbA1c (%)	3.0 ± 0.0	6.9 ± 0.6***
Fasting blood glucose (mM)	3.4 ± 0.3	6.7 ± 1.8***
Fed blood glucose (mM)	7.8 ± 0.5	25.2 ± 2.9***
Fasting insulin (ng/ml)	0.3 ± 0.1	2.1 ± 0.6***
Fed insulin (ng/ml)	1.5 ± 0.7	1.5 ± 0.3

***$P < 0.001$. HbA1c, glycated haemoglobin A1c.

Supplementary Table S2. Clinicopathological characteristics of PDAC patients

	non-T2D group (n=24)	T2D group (n=21)	*P* value
Age (year)*	70.5 (49-84)	71.0 (56-84)	0.699
Gender, n (%)			
Male	7 (29)	9 (43)	
Female	17 (71)	12 (57)	0.080
Smoker, n (%)	8 (33)	12 (57)	0.140
Body mass index (kg/m^2)*	22.7 (21.0-29.5)	22.3 (20.8-26.6)	0.991
HbA1c	6.0 (5.5-6.2)	7.6 (6.6-8.0)	< 0.001
Tumor size (mm)	29.5 (25-45)	30 (25-40)	0.864
T stage:			
T1-T2	0 (0)	1 (5)	
T3-T4	24 (100)	20 (95)	0.467
N stage:			
N0	9 (38)	10 (48)	
N1	15 (62)	11 (52)	0.555
Hisological grade:			
well-mod	19 (79)	17 (81)	
Por	5 (21)	4 (19)	1
ly-factor			
0-1	12 (50)	11 (52)	
2-3	12 (50)	10 (48)	1
v-factor			
0-1	4 (17)	3 (14)	
2-3	20 (83)	18 (86)	1
ne-factor			
0-1	4 (17)	3 (14)	
2-3	20 (83)	18 (86)	1
Location			
Ph	13 (54)	13 (62)	
Pb-t	10 (42)	6 (29)	
Phbt	1 (4)	2 (9)	0.569
Type of resection, n (%)			
Pancreatico-duodenectomy	11 (46)	13 (62)	
Distal pancreatectomy	10 (42)	6 (29)	

Total pancreatectomy	3 (12)	2 (9)	0.573
Neo-adjuvant chemotherapy, n (%)	1 (4)	4 (19)	0.169
Post-operative chemotherapy, n (%)	16 (67)	14 (67)	1
CA19-9 (U/mL)	56.0 (21.5 - 291.8)	102.0 (26.0 - 322.0)	0.682

*Median, PDAC, pancreatic ductal adenocarcinoma: T2D, type 2 diabetes mellitus: well-mod, well to moderately differentiated adenocarcinoma: Por, poorly differentiated adenocarcinoma: CA19-9, carbohydrate Antigen 19-9.

Supplementary Table S3. Univariate analysis of relapse-free and overall survival in PDAC subjects

Factor	RFS		OS	
	Median RFS (date)	*P* value	Median OS (date)	*P* value
Age (yrs): < 65 vs ≥ 65	1178 vs 441	0.231	NA vs 933	0.155
Male vs Female	446 vs 535	0.702	632 vs 1097	0.413
Tumor size (mm): < 30 vs ≥ 30	602 vs 281	0.24	1097 vs 597	0.052
N: (-) vs (+)	750 vs 407	0.0764	1266 vs 979	0.284
HbA1c (%): < 6.5 vs ≥ 6.5	638 vs 446	0.229	933 vs NA	0.180
T2D: (-) vs (+)	441 vs 638	0.046	632 vs NA	< 0.01
CA19-9 (U/mL): < 37 vs ≥ 37	946 vs 407	0.163	1266 vs 933	0.341
PSCa-L/CXCL13^{+} vs others	1212 vs 441	< 0.01	NA vs 847	< 0.01

PDAC, pancreatic ductal adenocarcinoma: RFS, relapse-free survival: OS, overall survival: NA, not applied: HbA1c, glycated haemoglobin A1c: T2D, type 2 diabetes mellitus: CA19-9, carbohydrate antigen 19-9: PSCa, pancreatic stellate cell activation score: PSCa-L, PSCa-low.

Supplementary Table S4. Multivariate analysis of overall and recurrence-free survival in PDAC subjects

Factor	RFS			OS		
	Risk ratio	95%CI	*P* value	Risk ratio	95%CI	*P* value
T2D: (-) vs (+)	1.476	0.66 - 3.29	0.341	2.443	0.81 – 7.33	0.111
PSCa-L/CXCL13^{+} vs others	0.352	0.13 - 0.93	0.034	0.269	0.07 – 1.03	0.056

RFS, recurrence-free survival: OS, overall survival: CI, confidence interval: T2D, type 2 diabetes mellitus: PSCa, pancreatic stellate cell activation score: PSCa-L, PSCa-low.

Supplementary Table S5. List of antibodies used in this study

Antibody name	Species	Catalog No.	Vendor	Experiments	Dilutions
M6a	Mouse	D055-3	MBL Life Science (Tokyo, Japan)	ICC	1:200
PTN	Rabbit	27117-1-AP	Proteintech (IL, USA)	ICC	1:200
PDGFRα	Rabbit	EPR22059-270	Abcam (Cambridge, UK)	ICC	1:200
Sca1	Rat	E13 161-7	Abcam (Cambridge, UK)	IHC, ICC	1:1000
CXCL13	Goat	AF472	R&D systems (MN, USA)	IHC, ICC, IF	1:100
Ly-6c	Rat	HK1.4	BioLegend (CA, USA)	IHC, ICC	1:100
αSMA	Rabbit	EPR5368	Abcam (Cambridge, UK)	IHC, ICC, IF	1:200
FABP4	Rabbit	EPR3579	Abcam (Cambridge, UK)	IHC, ICC, IF	1:200
CD31	Rabbit	D8V9E	Cell Signaling Technology, Inc., (MA, USA)	IHC	1:100
Ki67	Rabbit	ab66155	Abcam (Cambridge, UK)	IHC	1:200
CD3	Mouse	F7.2.38	Agilent (CA, USA)	IHC	1:200
CD8	Mouse	C8/144B	Agilent (CA, USA)	IHC	1:200
B220	Rat	RA3-6B2 (RUO)	BD Bioscience (NJ, USA)	IHC	1:200
CD45	Rabbit	20103-1-AP	Proteintech (IL, USA)	IHC	1:200
CD20	Mouse	L26	Leica (Wetzlar, Germany)	IHC	1:200
CXCL13	Goat	AF801	R&D systems (MN, USA)	IHC	1:100
F4/80-PE/cy7	Rat	BM8	BioLegend (CA, USA)	FACS	1:100
CD326 (Ep-CAM)-FITC	Rat	G8.8	BioLegend (CA, USA)	FACS	1:100
Ly6c-FITC	Rat	HK1.4	BioLegend (CA, USA)	FACS	1:50
Sca1-PE	Rat	D7	BioLegend (CA, USA)	FACS	1:200

Sca1-APC	Rat	D7	BioLegend (CA, USA)	FACS	1:200
Sca1-PE	Rat	D7	Thermo Fisher Scientific (MA, USA)	FACS	1:100
CXCL13-APC	Rat	DS8CX13	Thermo Fisher Scientific (MA, USA)	FACS	1:50
FITC-isotype	Rat	RTK2758	Biolegend (CA, USA)	FACS	1:100
APC-isotype	Rat	eBR2a	Biolegend (CA, USA)	FACS	1:100
PE-isotype	Rat	eBR2a	Biolegend (CA, USA)	FACS	1:100
PE/Cy7-isotype	Rat	RTK2758	Biolegend (CA, USA)	FACS	1:100
Alexa fluore 488 anti-mouse IgG (H+L)	Donkey	A21202	Thermo Fisher Scientific (MA, USA)	IF	1:500
Alexa fluore 594 anti-mouse IgG (H+L)	Donkey	A21203	Thermo Fisher Scientific (MA, USA)	IF	1:500
Alexa fluor 488 anti-rabbit IgG (H+L)	Donkey	A32790	Thermo Fisher Scientific (MA, USA)	IF	1:500
Alexa fluor 594 anti-rabbit IgG (H+L)	Donkey	A21207	Thermo Fisher Scientific (MA, USA)	IF	1:500
Alexa fluor 488 anti-gout IgG (H+L)	Donkey	A11055	Thermo Fisher Scientific (MA, USA)	IF	1:500

ICC, immunocytochemistry: IHC, immunohistochemistry: FACS, fluorescence assisted cell sorting: IF, immunofluorescence.

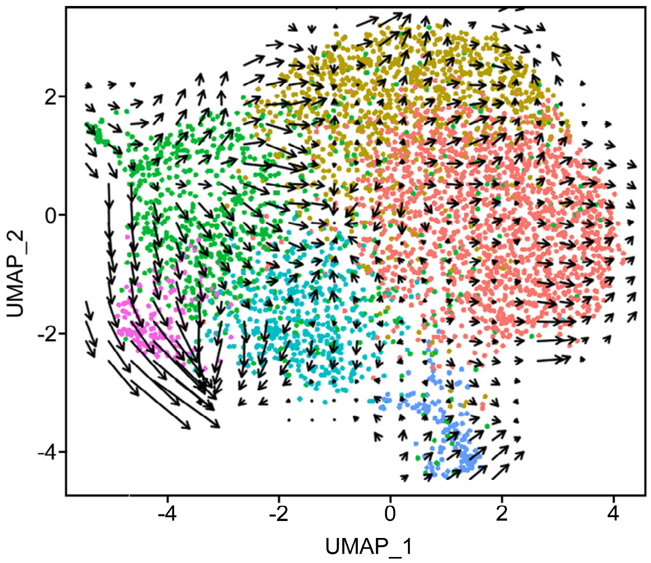

Supplementary Figure S1. RNA velocity analysis of Pancreatic fibroblasts.

Arrow direction and length indicate probable lineage trajectory and velocity, respectively.

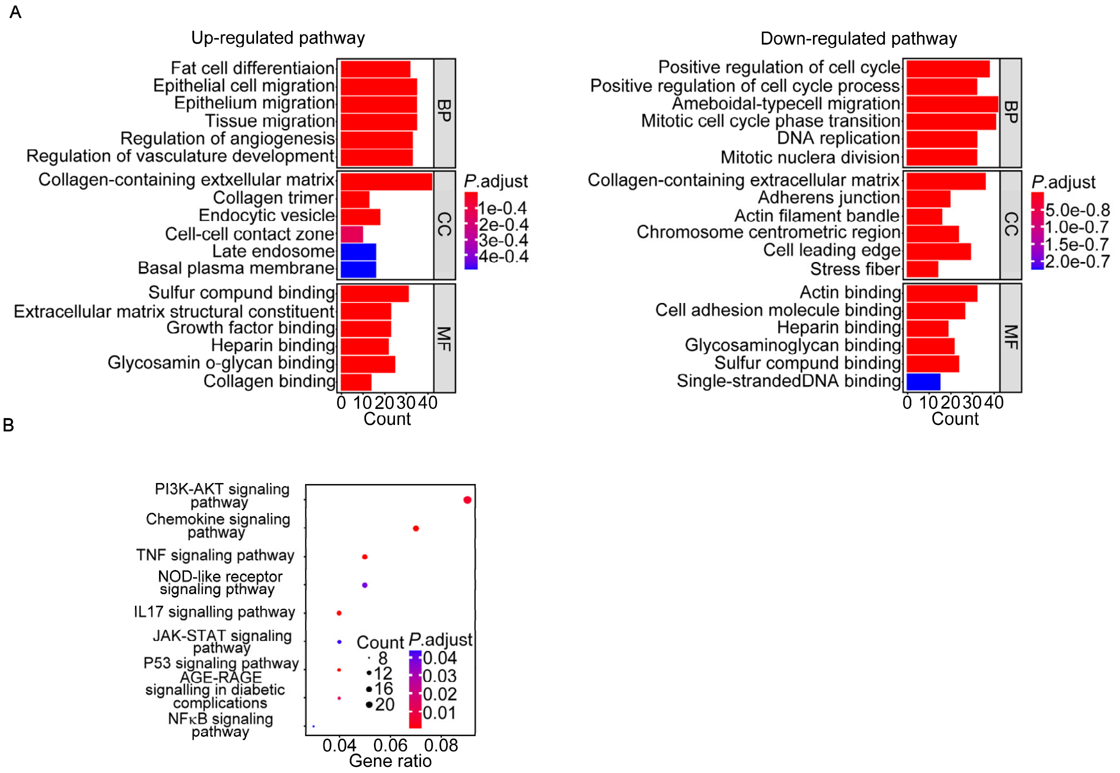

Supplementary Figure S2. Gene Ontology analysis and enriched analysis of tapPafs.

(A) Gene Ontology analysis of tapPafs revealed upregulated and downregulated processes. **(B)** Bar plots showing KEGG pathways enriched in tapPafs. Pafs, pancreatic fibroblasts; tapPafs, tumor immunity- and angiogenesis-promoting Pafs; KEGG, Kyoto Encyclopedia of Genes and Genomes; BP, biological process; CC, cellular component; MF, molecular function.

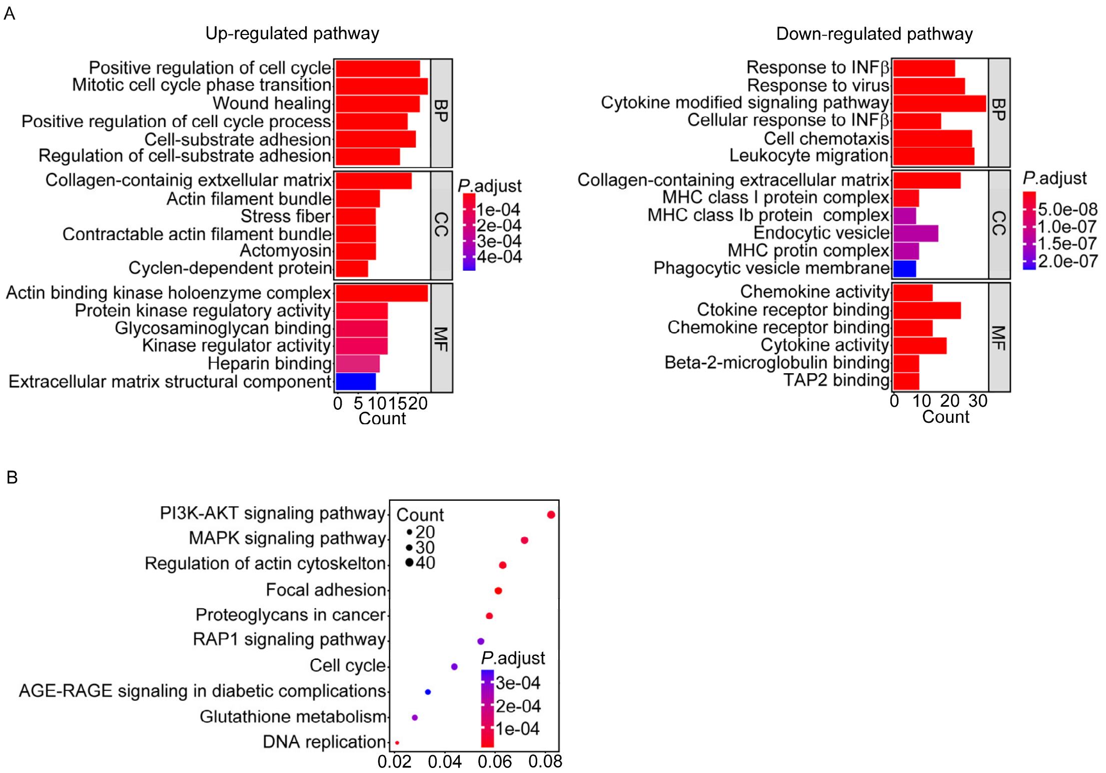

Supplementary Figure S3. Gene Ontology analysis and enriched analysis of myPafs.

(A) Gene Ontology analysis of myPafs revealed strong enrichment of upregulated pathways and downregulated pathways. **(B)** Bar plots showing KEGG pathways enriched in myPafs. myPafs, myofibroblastic Pafs; tapPafs, tumor immunity- and angiogenesis-promoting Pafs; KEGG, Kyoto Encyclopedia of Genes and Genomes; BP, biological process; CC, cellular component; MF, molecular function.

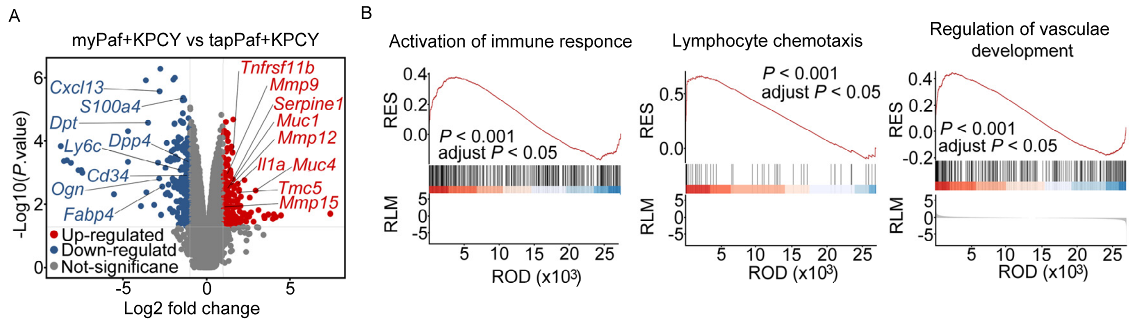

Supplementary Figure S4. Volcano plots and gene set enrichment analysis of tapPafs.

(A) Volcano plots illustrating the log2-fold changes and corresponding *P* values for differentially expressed genes in the tumor expression dataset between KPCY tumor cells cotransplanted with myPafs and those cotransplanted with tapPafs; $P < 0.01$ was considered to indicate statistical significance. **(B)** GSEA was performed on tumors transplanted with tapPafs or myPafs. Pafs, pancreatic fibroblasts; tapPafs, tumor immunity- and angiogenesis-promoting Pafs; myPafs, myofibroblastic Pafs; GSEA, gene set enrichment analysis.

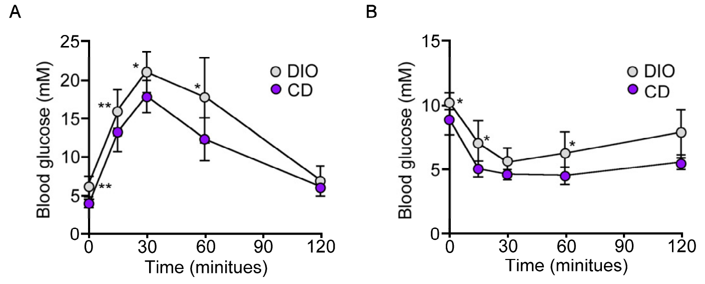

Supplementary Figure S5. OGTT and ITT of KPCY cell-transplanted mice.

(A) OGTTs were performed on KPCY cell-transplanted mice after 8 weeks of high-fat diet feeding (n=7 per each group). **(B)** The ITT was performed at the same time as the OGTT (n=7 per each group). OGTT, oral glucose tolerance test; ITT, insulin tolerance test; DIO, diet-induced obesity; CD, control diet. The data are presented as the mean ± SD. Statistical analysis was performed by 2-way ANOVA with post hoc multiple-comparison tests. $^{*}P < 0.05$, $^{**}P < 0.01$.

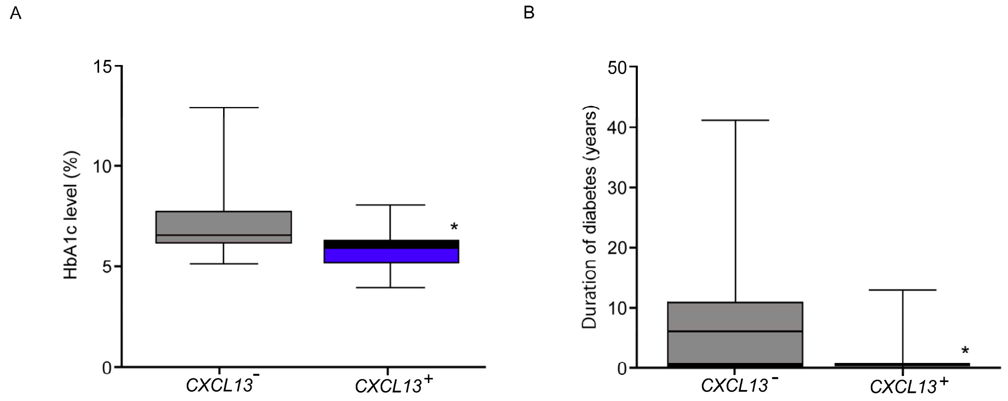

Supplementary Figure S6. Correlation between presence of *CXCL13*$^+$ stromal cells and diabetic factors. (**A**) HbA1c level in subjects with the presence of *CXCL13*$^+$ stromal cells was significantly higher than that in subjects with absence of *CXCL13*$^+$ stromal cells. (**B**) Duration of diabetes in subjects with the presence of *CXCL13*$^+$ stromal cells was significantly longer than in subjects with absence of *CXCL13*$^+$ stromal cells. Box and whiskers are median and 25% interquartile intervals. Bar means standard deviation. The data are presented as the mean ± SD. Statistical analysis was performed by Fisher's exact test and Mann – Whitney U test. $^*P < 0.05$.

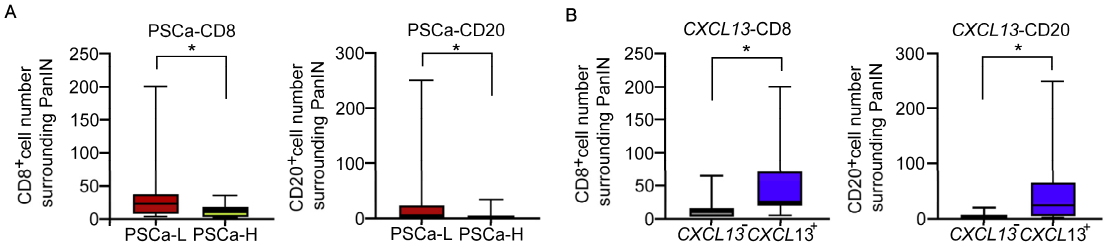

Supplementary Figure S7. CD8- and CD20-positive cells infiltration surrounding PanIN lesions of human PDAC patients.

(A) The density of CD8- and CD20-positive cells surrounding PanIN lesions quantitatively evaluated in immunostained sections according to the differences in the PSCa score (n=19; PSCa-H and n=26; PSCa-L) and **(B)** number of *CXCL13*-positive stromal cells (n=31; *CXCL13*$^{-}$ and n=14). PDAC, pancreatic ductal adenocarcinoma; PanIN, pancreatic intraepithelial neoplasia; PSCs; pancreatic stellate cells; PSCa, pancreatic stellate cell activation; PSCa-L, PSCa-low; PSCa-H, PSCa-high. Box and whiskers are median and 25% interquartile intervals. The data are presented as the means ± SD. Statistical analysis was performed by Fisher's exact test and Mann – Whitney U test. For multiple comparisons, the Z test with Bonferroni adjustment was used. Bar represents mean±SD. $^{*}P < 0.05$.

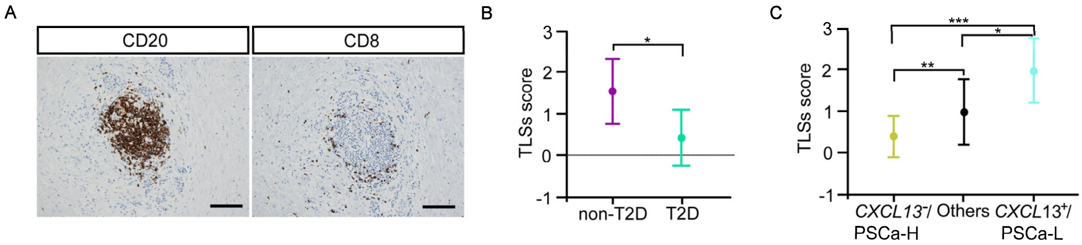

Supplementary Figure S8. Tertiary lymphoid structures in human PDAC samples with T2D.

(A) Density of TLSs quantitatively evaluated between non-T2D and T2D samples. **(B)** Density of TLSs quantitatively evaluated based on the number of *CXCL13*-positive stromal cells and the PSCa score. PDAC, pancreatic ductal adenocarcinoma; TLSs, tertiary lymphoid structures; T2D, type 2 diabetes; PSCs; pancreatic stellate cells; PSCa, pancreatic stellate cell activation; PSCa-L, PSCa-low; PSCa-H, PSCa-high. Statistical analysis was performed by Fisher's exact test and Mann – Whitney U test. Bar represents mean ± SD. $^{*}P < 0.05$, $^{**}P < 0.01$, $^{***}P < 0.001$. The scale bar represents 100 µm.
